# Kristine M. Alpi, AHIP, FMLA, Medical Library Association President, 2021–2022

**DOI:** 10.5195/jmla.2021.1387

**Published:** 2021-10-01

**Authors:** Patricia E. Gallagher

**Affiliations:** 1 patriciaegallagher@gmail.com

## Abstract

In this profile, Kristine M. Alpi, AHIP, FMLA, Medical Library Association (MLA) president, 2021–2022, is described as committed to public health, professional development, and the growth and evolution of MLA. She teaches and speaks on the shared health impact from interactions among animals, humans, and the environment, and she mentors graduate students and fellows in librarianship and informatics. Alpi earned her PhD in educational research and policy analysis in 2018 and directs the Oregon Health & Science University Library.

**Figure:**
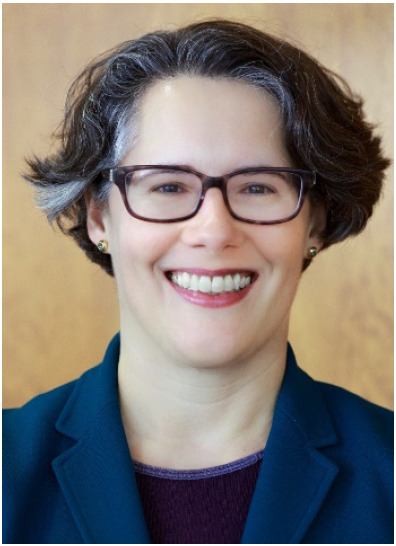


It's a distinct honor to be able to tell you about the career of Kristine Markovich Alpi, Medical Library Association (MLA) president for 2021–2022.

I first met Kris when she arrived at the New York Academy of Medicine, where she was starting a job as education coordinator for what was then the Region 1 Regional Medical Library. She had, however, already begun preparing herself for excellence in library services, having worked as a hospital librarian in Indiana and then participating in the National Library of Medicine (NLM) Associate Fellowship Program.

Once settled in New York, Kris pursued her master's in public health, enrolling in the Hunter College School of the Health Professions. After working as an information services librarian and lecturer at the Weill Cornell Medical College, she took on the position of library manager at the New York City Department of Health & Mental Hygiene's Public Health Library, where she directly served the public health professionals that served the largest city in the United States. She also continued as a lecturer in public health at Weill Cornell, teaching students in evidence-based medicine, epidemiology, and biostatistics.

With her relocation to North Carolina as director of the William R. Kenan, Jr. Library of Veterinary Medicine at North Carolina State University (NCSU), Kris entered a new area of public health—that of the shared health impact from interactions among animals, humans, and the environment. Her recent coauthored article that appeared in the NLM's Director's Blog outlines the importance of One Health—these shared public health impacts [1]. She continued to teach, now emphasizing the place of animals in the public health universe. She also began work on her PhD in educational research and policy analysis from NCSU, which she completed in 2018.

December 2018 began a new phase in Kris's career as she moved to Portland and assumed the directorship of the Oregon Health & Science University Library. As part of her responsibilities as university librarian and associate professor in the Department of Medical Informatics & Clinical Epidemiology, she still educates students on informatics and epidemiology and serves as a mentor to graduate students and fellows.

Kris's work in public health has extended to educating consumers by locating accurate and timely web-based information. From 1998 to 2009, she used her expertise in Spanish to build the Spanish side of the bilingual web portal NOAH (New York Online Access to Health). After grant funding ceased, NOAH became a volunteer-driven project—Kris managed the Spanish content, as well as volunteering to work on the redesign committee so that the new interface was user-friendly to Spanish speakers. For that work, she was one of the awardees when NOAH was given the Thomson Scientific/Frank Bradway Rogers Information Advancement Award in 2006.

MLA has benefited from Kris's service. She has been a member of the Academy of Health Information Professionals (AHIP) since 1997. She served on the National Program Committee three times and has been elected to the Nominating Committee twice and to the MLA Board. As a member and eventual chair of the Public Health and Health Administration Section (now Caucus), Kris worked with a committee to create a comprehensive list of Medical Subject Headings (MeSH) that would benefit searching for the public health community; many of these terms have been added to the MeSH vocabulary. She also chaired the Research Caucus and served on the editorial board of the *Journal of the Medical Library Association*. In 2021, Kris was selected as a Fellow of MLA.

I look forward to Kris Alpi's presidential year. Her commitment to professional development and to the growth and evolution of MLA will benefit all members. Please join me in welcoming her to her new position.

## References

[1] Alpi KM, Johnson T, Moberly KM. All for one…health for all: the role of open access, evidence-based information to improve health for all species [Internet]. Musings from the Mezzanine. 13 Oct 2020 [accessed 19 Aug 2021]. <https://nlmdirector.nlm.nih.gov/2020/10/13/all-for-one-health-for-all-the-role-of-open-access-evidence-based-information-to-improve-health-for-all-species/>.

